# Supplementary material for: People who inject drugs in metropolitan Chicago: A meta-analysis of data from 1997-2017 to inform interventions and computational modeling toward hepatitis C microelimination
Source: PLoS One. 2022 Jan 12;17(1):e0248850. doi: 10.1371/journal.pone.0248850 (PMC8754317; doi:10.1371/journal.pone.0248850)
Supplement: S2 File — Figures showing estimates for proportions of PWID reporting sharing of ancillary injection equipment (S1 Fig), syringe-mediated drug sharing (S2 Fig), and obtaining syringes from a syringe service program (S3 Fig). (PDF) [file pone.0248850.s002.pdf]

People who inject drugs in metropolitan Chicago: A meta-analysis of data from 1997-2017 to inform interventions and computational modeling toward hepatitis C microelimination

Supplemental Material

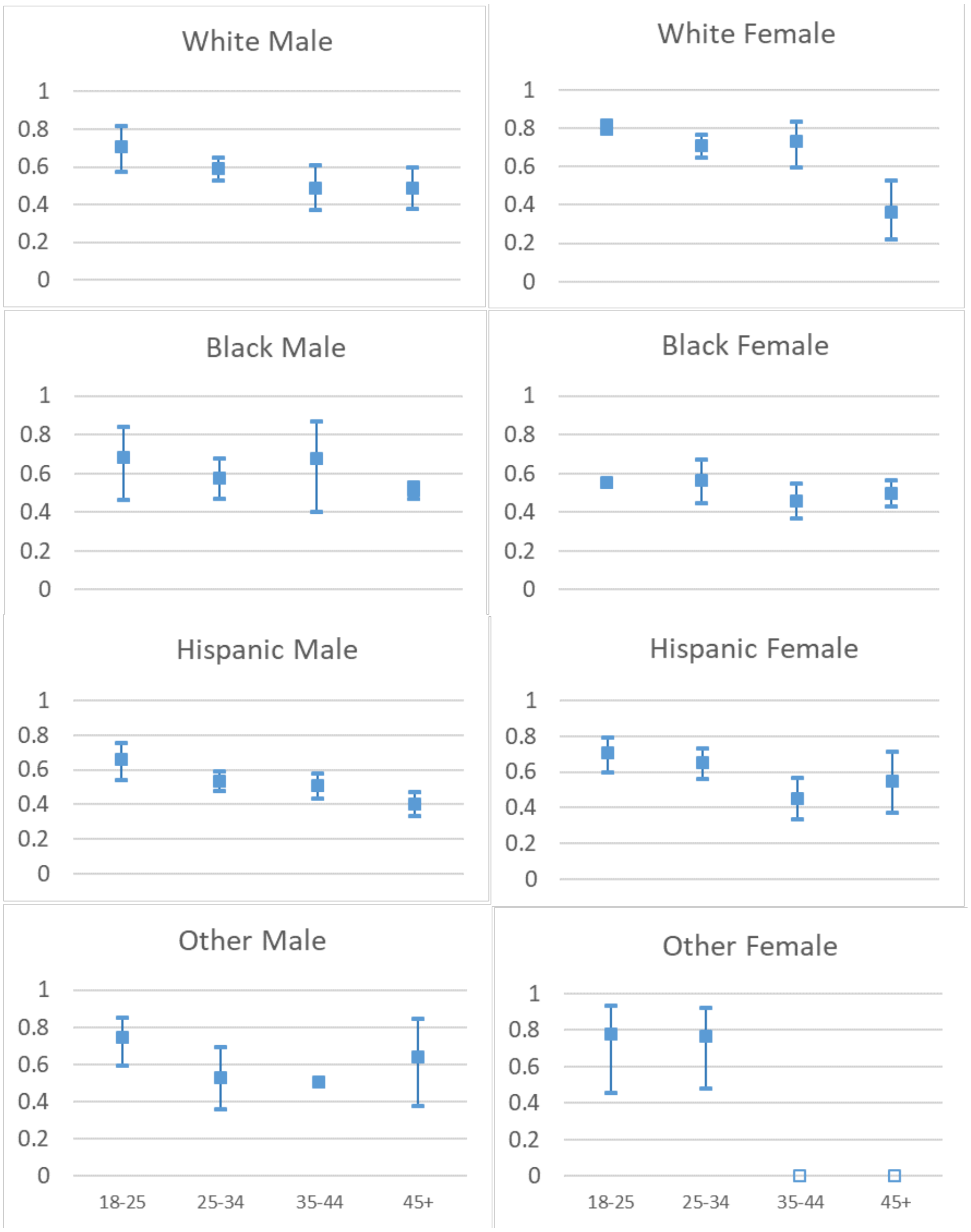

Fig S1. Proportions of PWID reporting equipment sharing

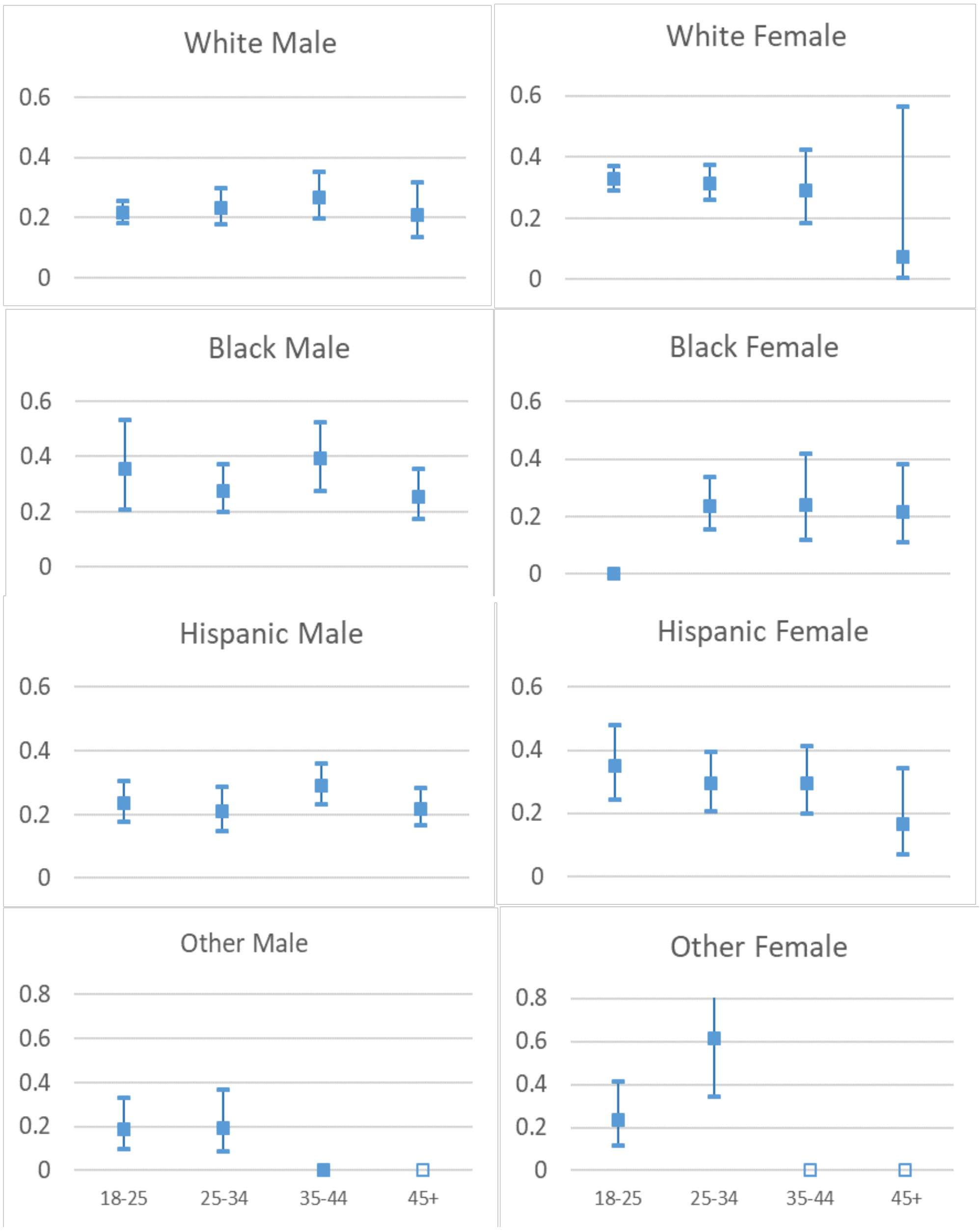

Fig S2. Proportions of PWID reporting syringe mediated drug sharing

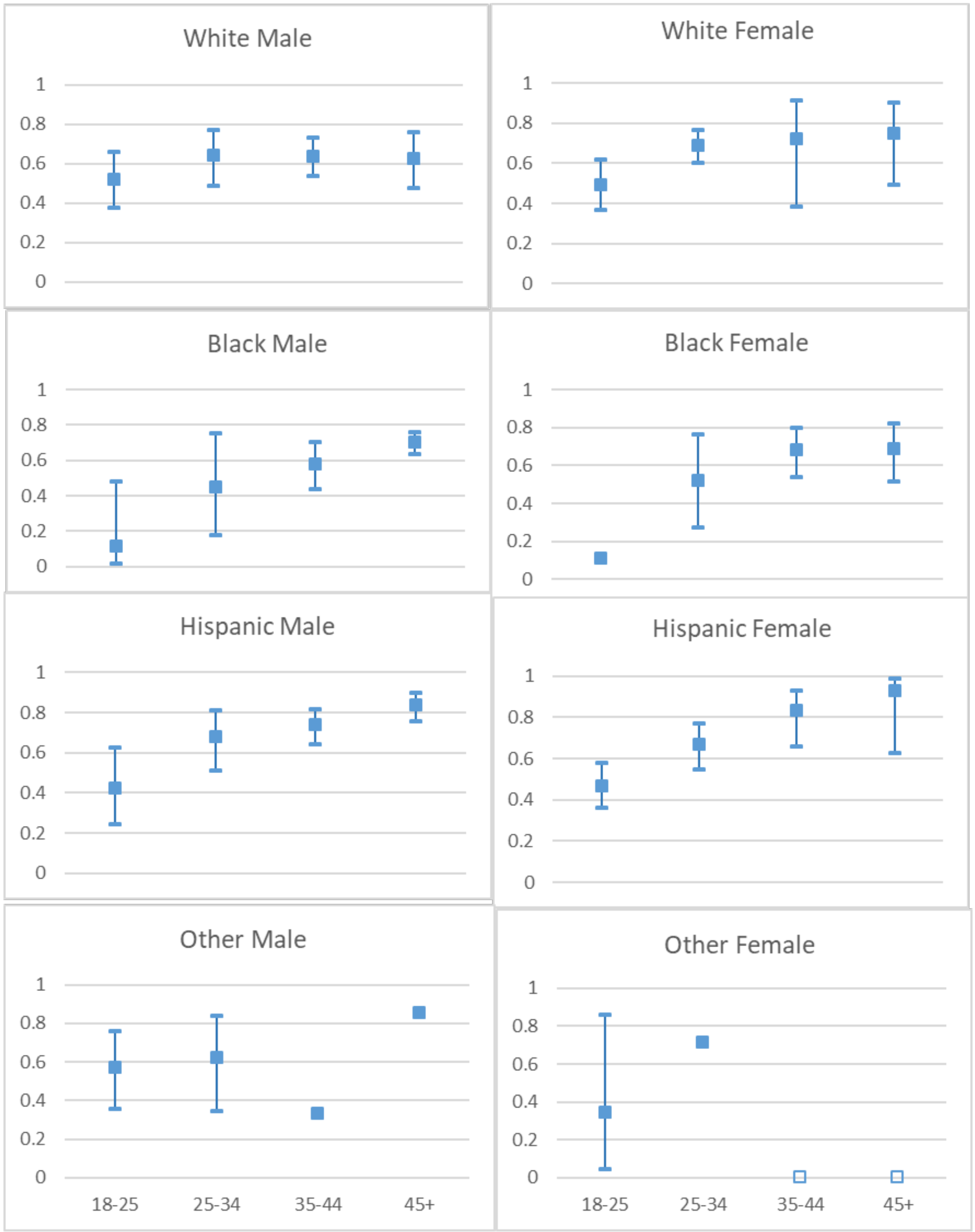

Fig S3. Proportions obtained syringes from a syringe exchange program
