# Supplementary material for: People who inject drugs in metropolitan Chicago: A meta-analysis of data from 1997-2017 to inform interventions and computational modeling toward hepatitis C microelimination
Source: PLoS One. 2022 Jan 12;17(1):e0248850. doi: 10.1371/journal.pone.0248850 (PMC8754317; doi:10.1371/journal.pone.0248850)
Supplement: S1 Table — (PDF) [file pone.0248850.s003.pdf]

People who inject drugs in metropolitan Chicago: A meta-analysis of data from 1997-2017 to inform interventions and computational modeling toward hepatitis C microelimination

Table S1. Random effects meta-analysis estimates of characteristics of PWID population.

Parameter	# studies	Estimate	CI lower	CI upper	tau^2	tau	PI lower	PI upper	I^2
**Proportions**									
injects alone	8	0.13	0.078	0.218	0.694	0.833	0.017	0.574	0.964
RSS	13	0.31	0.247	0.377	0.300	0.548	0.113	0.610	0.987
DSS	5	0.39	0.346	0.444	0.047	0.216	0.232	0.583	0.919
SMS	11	0.25	0.216	0.284	0.076	0.276	0.146	0.390	0.881
shared equipment	9	0.63	0.562	0.687	0.155	0.394	0.385	0.818	0.962
homeless	10	0.38	0.280	0.487	0.498	0.706	0.099	0.770	0.977
exchange sex	11	0.18	0.137	0.229	0.251	0.501	0.062	0.416	0.936
current drug treatment	10	0.13	0.096	0.166	0.228	0.477	0.044	0.317	0.938
SSP	10	0.59	0.455	0.706	0.704	0.839	0.157	0.915	0.983
employed (FT/PT)	10	0.19	0.092	0.358	1.868	1.367	0.009	0.866	0.991
any paid work	10	0.43	0.258	0.628	1.600	1.265	0.035	0.942	0.994
heterosexual	9	0.85	0.807	0.884	0.184	0.428	0.658	0.943	0.971
gay/homosexual	9	0.03	0.018	0.047	0.478	0.692	0.005	0.145	0.925
bisexual	9	0.11	0.074	0.154	0.364	0.604	0.026	0.354	0.977
MSM	12	0.05	0.035	0.080	0.501	0.708	0.011	0.226	0.958
injected heroin by itself	13	0.98	0.954	0.987	1.142	1.069	0.771	0.998	0.966
injected cocaine	10	0.36	0.307	0.406	0.108	0.329	0.198	0.551	0.927
injected speedball	11	0.24	0.185	0.301	0.272	0.522	0.083	0.519	0.943
injected methamphetamine	13	0.03	0.018	0.040	0.456	0.675	0.006	0.117	0.904
**Normal means**									
income	6	1367	1132	1603	81976	286	505	2229	0.971
age first injected	13	22.1	20.95	23.24	4.37	2.09	17.32	26.87	0.993
years injected	12	9.89	6.65	13.14	32.76	5.72	-	23.17	0.998
injection frequency	13	75.1	60.5	89.8	698.6	26.4	14.7	135.6	0.996
percent times RSS	10	0.12	0.090	0.153	0.002	0.049	0.004	0.239	0.957

percent times share equip.	8	0.28	0.228	0.327	0.005	0.070	0.096	0.459	0.962
percent times SMS	8	0.08	0.070	0.097	0.000	0.017	0.040	0.127	0.744
network pct male	6	0.66	0.644	0.677	0.000	0.017	0.608	0.713	0.756
network pct white	4	0.54	0.278	0.799	0.070	0.265	-	-	0.998
network pct black	4	0.23	0.050	0.404	0.032	0.180	-	-	0.997
network pct hispanic	4	0.20	0.103	0.288	0.009	0.093	-	-	0.982
network pct young	3	0.40	0.053	0.742	0.092	0.304	-	-	0.998
network pct city	3	0.62	0.379	0.860	0.045	0.211	-	-	0.995
**Poisson means†**									
# sex partners	13	6.68	4.19	9.16	20.84	4.57	-	17.11	0.999
# exchange partners	5	7.47	2.84	12.10	27.84	5.28	-	25.87	1.000
IDU network[a]	6	16.51	10.12	22.90	63.80	7.99	-	40.46	1.000
injection network[b]	5	4.27	3.38	5.16	1.02	1.01	0.74	7.79	0.993
# people RSS[c]	9	2.15	1.64	2.67	0.60	0.78	-	4.09	0.981
# people DSS[c]	5	2.42	1.86	2.98	0.39	0.62	-	4.60	0.978
# people share equipment[c]	6	3.49	2.57	4.40	1.29	1.13	-	6.89	0.989

RSS: receptive syringe sharing; DSS: distributive syringe sharing; SMS: syringe mediated sharing; SSP: syringe service program; CI: 95% confidence interval; PI: 95% prediction interval; tau^2: estimated between study variance; tau: estimated standard deviation of true parameter; I^2: proportion of variation in estimate attributable to heterogeneity

a Number of people you know who inject drugs; b Number of people you injected with; c Among people who shared

† Poisson standard deviation was used in analysis of count variables. Estimates of between-study variance (tau2, I2) may be inflated.

- prediction intervals suppressed when values are out of range

Note: Prediction intervals are a popular way of expressing the amount of heterogeneity in a meta-analysis (Riley et al 2011). However, they can be very problematic when the number of studies is small, in which case they can appear spuriously wide or spuriously narrow (Deeks et al. 2019). They can also produce values that are out of the possible range for count variables (minimum zero or 1), or proportions (range 0 to 1).

Riley RD, Higgins JPT, Deeks JJ. Interpretation of random effects meta-analyses. BMJ 2011; 342: d549.

Deeks JJ, Higgins JP, Altman DG, Cochrane Statistical Methods Group. Analysing data and undertaking meta-analyses. In: Higgins JPT, Thomas J, Chandler J, Cumpston M, Li T, Page MJ, Welch VA, editors. Cochrane Handbook for Systematic Reviews of Interventions. 2nd ed. Chichester, UK: John Wiley & Sons; 2019. p. 241-84.
